# Social Cohesion, Mental Well-Being, and the Role of Smart Technology and Pet Ownership Among Social Housing Residents: Cross-Sectional Cohort Study

**DOI:** 10.2196/75451

**Published:** 2025-10-24

**Authors:** Emmylou Rahtz, Andrew James Williams, Timothy J Taylor

**Affiliations:** 1 European Centre for Environment and Human Health, Faculty of Health and Life Sciences, University of Exeter, Peter Lanyon, Penryn Campus, Penryn, TR10 9FE, United Kingdom, 44 1392 661000; 2 Scottish Collaboration for Public Health Research and Policy, School of Health in Social Science, University of Edinburgh, Edinburgh, United Kingdom

**Keywords:** mental well-being, social cohesion, smart technology, gaming, pet ownership, social housing

## Abstract

**Background:**

Smart technology has been shown to have varied effects on social cohesion and mental well-being. There has been very little research on associations between pet ownership and social cohesion and mental well-being.

**Objective:**

This study aimed to explore associations between social cohesion and mental well-being and ownership of different forms of smart technology, dogs, and cats in a sample of adult social housing occupants in Cornwall, United Kingdom.

**Methods:**

This was a cross-sectional study that collected data on people’s living environment, health, and well-being, including the Short Warwick-Edinburgh Mental Wellbeing Scale and an 8-item social cohesion scale. Participants were social housing residents in Cornwall in the South West of the United Kingdom. We used cross-sectional regression analyses to explore associations between people’s ownership of different forms of smart technology and pets, and their reported levels of social cohesion and mental well-being.

**Results:**

There were no statistically significant associations between social cohesion and ownership of either smart technology or pets. Unadjusted regressions for mental well-being showed an association with owning a smartphone. However, after adjusting for age, gender, and socioeconomic status, this effect was no longer significant. Those who owned any smart technology (*b*=1.76, 95% CI 0.06-3.45; *P*=.04) and those who owned a games console (*b*=2.39, 95% CI 0.59-4.19, *P*=.01) had significantly higher levels of mental well-being, after adjusting for age, gender, and socioeconomic status; the effect held after social cohesion was added to the model. Counterintuitively, owning two or more dogs was associated with lower levels of mental well-being (*b*=−2.12, 95% CI −4.06 to −0.19; *P*=.03) compared with owning no dogs, after adjusting for age, gender, socioeconomic status, and social cohesion. However, there were no significant differences in mental well-being related to cat ownership.

**Conclusions:**

Previous research suggests that the beneficial effects of smart technology are context-dependent, and our results support that. While we did not observe significant effects on social cohesion, owning any smart technology or a games console specifically was associated with well-being benefits. There are limited data on pet ownership and social cohesion or mental well-being: our data suggest there is no strong relationship with social cohesion but owning multiple dogs can have negative effects.

## Introduction

### Social Cohesion and Well-being

The costs of mental ill health in England have been estimated at £119 billion (US $161 billion) per annum [1]. A number of factors have been found to be linked to subjective well-being, including health status, housing tenure, relationship status, and age [2].

Social cohesion describes the extent to which people feel integrated within their neighborhood, based on shared values and a sense of solidarity [3]; it has also been shown to be a powerful indicator of subjective well-being [3], and cross-national studies indicate that the United Kingdom’s position in national rankings of social cohesion is falling [4].

The Smartline project recruited a sample of people living in social housing and included measures on social cohesion, mental well-being, physical and mental health, as well as physical measures of the home environment. Social housing is rented accommodation costing below the market rate, typically offered to people with specific health needs or in particular economic circumstances by not-for-profit organizations. The project was based in the United Kingdom county of Cornwall. Cornwall has been in receipt of initiatives to increase digital inclusion in rural areas, with decreased social isolation and improved access to health care among the aims [5].

Previous research among the Smartline cohort showed that social cohesion was associated with mental well-being, but not with physical or mental health [6], and gave rise to further questions about the role of smart technology and pet ownership.

### Smart Technology, Social Cohesion, and Well-being

Qualitative studies suggest that smart technologies and, more broadly, internet and communications technologies (ICT) can have a positive impact on social cohesion and societal capabilities [7]. Smart technology initiatives can provide tools for inclusive growth: early initiatives in digital inclusion led to social and economic benefits in rural communities [8], and smartphones, used appropriately, can improve social connections among older adults [9]. In rural settings, it has been argued that ICT and smart technologies can encourage people to form social groups based on personal choice rather than geographical location, the so-called “disembedding” of social relationships [10]. Clearly, this can have both positive and negative effects, improving cohesion with online communities at the potential cost of geographically local connections. ICT allows people to work remotely, potentially reducing the potential for local integration [10]. Positive in-person social integration can be driven by “the commitment of the local ‘bohemian’ creative class to developing a sense of solidarity” [10], which would clearly be highly context dependent. Meanwhile, there is widespread societal concern about the effects of smartphone use on mental health, particularly among adolescents [11,12]. Some evidence supports this, indicating, for example, that problematic smartphone use among adolescents can be associated with higher anxiety [13], and spending less time with family and friends [14], both of which may affect sense of integration in real-world communities. However, equally, they may enable people to create stronger connections within the community they live in rather than with colleagues: rural isolation can make local social relationships even more important, and ICT can connect people in ever-evolving new ways [3]. ICT has been shown to have the potential to improve social well-being among older age groups [15]; however, this is a nuanced situation, with different age groups experiencing varied benefits from different applications of technology [16]. Smart technology and ICT are increasingly integral to society, particularly post pandemic, but the role they play can differ substantially depending on an area’s rurality, history, location, and socioeconomic makeup [10].

### Pets, Social Cohesion, and Well-Being

The limited body of research around pet ownership and social cohesion is constrained to dog ownership and suggests that small local parks, and specifically those used by dog owners, can contribute to neighborhood cohesion and ethnic integration [17], by facilitating regular, informal communication between dog walkers and other park users. However, Japanese researchers observed no association between dog walking and social cohesion and only a modest relationship with social capital [18]. Although acquiring a dog is associated with an increase in how much people walk, it does not affect their perception of their neighborhood’s social cohesiveness [19]. Dog ownership may explain the varying beneficial effects of living near green space, with dog owners more likely to meet recommended activity targets than others [20]. In turn, this is likely to have positive implications for mental well-being. Conversely, though, older adults may spend more time at home to avoid leaving dogs alone, thus reducing opportunities for physical activity and social interactions [21]. Having a dog can be a catalyst for casual interactions in public places, although this effect has been shown to be breed-specific, being stronger with “appealing” dogs like Labradors and weaker with Rottweilers [22]. A small study of older people with and without dogs found no difference in the length of conversations people engaged in while walking, although dog owners walked more frequently; however, dog owners reported higher levels of well-being, including regular socializing with friends and relatives [23]. Pet ownership is associated with positive physiological measures, including blood pressure and cholesterol levels, and with improved coping with chronic illness [24].

There is considerably less literature dealing specifically with cat ownership rather than dog ownership, in part because there are fewer potential mechanisms for effects on health, such as regularly walking in public places [25]. However, cats are associated with slightly lower caregiver burden than dogs, particularly in terms of ill animals [26]. Research also suggests personality differences between “dog people” and “cat people.” For example, an online study found that those who identified as “dog people” had higher levels of extraversion and agreeableness and lower levels of neuroticism than “cat people” [27]. Furthermore, research has shown that “cat people” score lower than “dog people” for factors including social boldness and warmth, and higher for self-reliance, indicating that they tend to be more individualistic, self-sufficient, and shy. This reinforces the popular perception that “cat people” are more introverted and supports the notion that dog ownership is associated with more casual social interactions [27], thereby enabling greater social cohesion [28]. We therefore wanted to explore the role of cat and dog ownership on social cohesion in this cohort.

The literature therefore suggests that both smart technology and pet ownership have potential associations with social cohesion, but these can be positive and negative and may be context dependent. Social cohesion itself appears to be context dependent, and there are growing calls for it to be studied at a local level to take account of this [6,29]. It is also a potentially modifiable factor influencing health [6], with the potential to be affected by technology or pets. We wanted to explore this in the relatively homogeneous cohort of the social housing communities [6] in the Smartline project.

This analysis sought to answer two questions: (1) Is there an association between the use of smart technology and levels of social cohesion or mental well-being in this cohort? (2) Is there an association between pet ownership and levels of social cohesion or mental well-being in this cohort?

We hypothesized that smart technology overall would be associated with lower levels of social cohesion, because it would improve distant rather than local communication. In contrast, we expected dog ownership to be associated with better social cohesion. We speculated that cat ownership would be associated with worse social cohesion, on the basis that owners might be more introverted and less integrated within their community. Because of the association between social cohesion and mental well-being already shown in this cohort [6], we planned to consider the role of mental well-being in these relationships.

## Methods

### Overview

This is a cross-sectional cohort study of the social cohesion and well-being of participants in the Smartline project. Smartline was a collaboration between the University of Exeter, the social housing provider Coastline Housing, the local government body, Cornwall Council, and the charity Volunteer Cornwall. The project ran from March 2017 to January 2023.

Coastline Housing residents in West Cornwall (Camborne, Pool, Illogan, and Redruth) were invited by Coastline Housing to take part in the project. Because these areas have a high concentration of Coastline Housing, communities could be studied as well as individual homes. Participants were identified and approached by Coastline Housing. Smartline sought to recruit approximately 350 households to meet the requirements of the wider project. Of 2000 Coastline households, 649 were approached and 329 consented to take part. They consented to have sensors installed in their homes and to take part in associated Smartline surveys. The sensors collected home environment data such as humidity, temperature, electricity usage, and carbon dioxide levels and are reported elsewhere [6,30]. This was a convenience sample with households approached on a street-by-street basis until enough households had been recruited. Participants were primarily recruited during the day and were paid a modest incentive of £10 (US $13.5); participants were excluded if they lacked the capacity to give informed consent.

Each recruited household had a main participant aged 18 years or more, who gave written informed consent. Main participants were subsequently interviewed for the survey in their homes at a time suitable for them (typically between 9 AM and 5 PM) by teams of 2 researchers from the University of Exeter. Researchers kept anonymized, contextual field notes of incidental observations and discussed these among themselves and the wider team as relevant. Baseline data were collected between September 2017 and April 2018 (n=303), with a booster sample collected between August and November 2018 (n=26).

The survey asked for sociodemographic information about the main participant, the age and gender of other household members, and detailed information about the participant’s home (including heating use, mold, damp, etc). The household’s ownership of different kinds of technology and pets was also collected to further understanding of social cohesion and mental well-being, as well as indoor air quality. Participants also completed standardized measures about their perception of social cohesion [31] and their own mental well-being (Short Warwick-Edinburgh Mental Wellbeing Scale [SWEMWBS]) [32]; these were the outcome variables for the analysis presented in this paper.

Both outcome measures were coded in accordance with published protocols [31,33] and were analyzed as continuous variables. If one or two items had been missed from the social cohesion scale’s 8 items or the SWEMWBS’s 7 items, those items were imputed using the mean of the other responses; where 3 or more items were missing, the total score for that measure was classified as missing.

Ownership of different forms of smart technology (any smart technology, smartphone, smartwatch, or games console) was analyzed dichotomously. The survey asked for the exact number of cats or dogs participants owned; for analysis, this was categorized as 0, 1, or 2 or more.

Participants’ postcodes were used to link the Index of Multiple Deprivation (IMD 2015) and rural-urban classification data to survey data. IMD or rural-urban classification data were not available for some properties, and they were therefore classified as missing.

Each cleaned, coded variable was summarized, and differences between those with and without complete data were tested.

The analyses presented are cross-sectional analyses of the baseline data. Sensitivity analyses on complete versus missing data used *t* tests, chi-square, Fisher exact, and Wilcoxon rank sum tests. We used univariate regressions to explore associations between the outcome variables of social cohesion and mental well-being, and the explanatory variables of age, gender, socioeconomic status (SES, comprising IMD decile, education, and employment status), ownership of smart technology, and pets. We then carried out multivariable regression to further explore associations for SWEMWBS. Analyses were performed using STATA 16 (StataCorp) statistical software [34]. We adhered to the STROBE (STrengthening the Reporting of OBservational studies in Epidemiology) statement checklist [35]

### Ethical Considerations

The University of Exeter Business School Research Ethics Committee, which conforms to the principles embodied in the Declaration of Helsinki, granted ethical approval for the Smartline project (eUEBS002996 v4.0). All participants provided written informed consent for the collection and analysis of their data. Data were anonymized. In total, 3 participants were potentially identifiable; their data were therefore treated as missing. Participants received modest financial compensation for the study.

## Results

Of 329 study participants, 308 (93.6%) provided complete data. A further 3 participants (3/329, 0.9%) were treated as missing because they were potentially identifiable.

There were no statistically significant differences between complete and missing cases in terms of age, gender, rural-urban classification, employment, social cohesion, mental well-being, ownership of smart technology, or pets. However, there were statistically significant differences in terms of IMD ranking and education. These data are shown in Table 1; meanwhile, Table S1 in the Multimedia Appendix 1 provides a description of all key variables.

**Table 1.** Participant characteristics of social housing residents in the Smartline cohort study in South West England, United Kingdom, 2017‐2018.

Variables	Complete^a^ (305)	Missing^a^ (24)	*P* value^b^
Age (years), mean (SD)	54.1 (17.6)	53.4 (14.2)	.87
Gender, n (%)			.91
Male	95 (31.2)	7 (30)	
Female	210 (68.9)	17 (70)	
IMD^c^ 2015			
Ranking	4446 (964‐8548)	1512 (964‐10,826)	.94
1st decile (most), n (%)	150 (49.2)	14 (56.5)	.001
2nd decile	47 (15.4)	2 (8.7)	
3rd decile	38 (12.5)	2 (8.7)	
4th decile	70 (23)	3 (13)	
5th decile	0 (0)	0 (0)	
6th decile	0 (0)	3 (13)	
7th decile	0 (0)	0 (0)	
8th decile	0 (0)	0 (0)	
9th decile	0 (0)	0 (0)	
10th decile (least)	0 (0)	0 (0)	
RUC^d^ 2011, n (%)			.61
Urban city and town	284 (93.1)	23 (95.8)	
Other	21 (6.9)	1 (4.2)	
Education, n (%)			.02
Secondary or primary	202 (66.2)	14 (57.9)	
Further	87 (28.5)	5 (21.1)	
Higher	16 (5.3)	5 (21.1)	
Employment, n (%)			.85
In work	60 (19.7)	4 (15)	
Education or training	7 (2.3)	0	
Retired	201 (33.4)	8 (35)	
Not at work	136 (44.6)	12 (50)	
Social cohesion, mean (SD)	26.9 (6.5)	26.1 (5.6)	.60
SWEMWBS^e^	24.1 (5.2)	23.3 (7.1)	.50
Any Smart tech, n (%)			
No	45 (14.8)	3 (12.5)	≥.99
Yes	260 (85.3)	21 (87.5)	
Smartphone (Yes)	213 (69.8)	14 (58.3)	.24
Smartwatch (Yes)	23 (7.5)	1 (4.2)	≥.99
Games console (Yes)	35 (11.5)	3 (12.5)	.74
Dogs			
0	201 (65.9)	12 (50)	.27
1	74 (24.3)	9 (37.5)	
2+	30 (9.8)	3 (12.5)	
Cats			
0	212 (69.5)	18 (75)	.45
1	52 (17.1)	5 (20.8)	
2+	41 (13.4)	1 (4.2)	

a Values are presented as mean (SD) where data are normally distributed, median (IQR) when data were skewed, and percentages for categorical data.

b *t* tests, chi-square, Fisher exact, and Wilcoxon rank sum (Mann-Whitney *U*) tests were used to compare complete and missing groups.

c IMD: Index of Multiple Deprivation.

d RUC: Rural Urban Classification.

e SWEMWBS: Short Warwick-Edinburgh Mental Wellbeing Scale.

Among complete cases, the mean age of participants was 54 years, and around two-thirds (210/305, 69%) were female. Almost half (150/305, 49.2%) lived in the poorest 10% of English postcodes according to IMD 2015 rankings, and all were in the poorest 40%. In total, 93% (284/305) were classified as living in an urban area. Two-thirds of participants (202/305, 66.2%) had left education after primary or secondary school, with just 1 in 20 (16/305, 5.3%) having completed higher education. In total, 1 in 5 participants were currently in work (60/305, 19.7%); one-third were retired (102/305, 33.4%), and more than two-fifths were currently not in work (136/305, 44.6%).

Social cohesion scores were compared with a sample of residents of Caerphilly, Wales, which had comparable levels of deprivation and which used the same measure of social cohesion [36]. At baseline, White et al’s [36] intervention group was categorized as having low, medium, or high levels of social cohesion, which we replicated in the Smartline data. White et al [36] reported 25.8% as low (Smartline 20/305, 6.6%), 36.6% as medium (Smartline 212/305, 69.5%), and 37.6% as high (Smartline 115/305, 23.9%). The mean Smartline score on SWEMWBS was 24.1 (SD 5.2); this is comparable with the UK population norm of 23.6 (SD 3.9), from the Health Survey for England 2011 [33]. A large majority (260/305, 85.3%) of the sample owned at least one form of smart technology in their household. Over two-thirds (213/305, 69.8%) had a smartphone; a smaller proportion owned a smartwatch (23/305, 7.5%) or games console (35/305, 11.5%). One-third of participants (104/305, 34.1%) had one or more dogs in the household, and slightly fewer (46/305, 30.5%) had one or more cats.

There were no statistically significant differences in social cohesion by age, gender, or socioeconomic variables. Nor were there differences by the variables of interest: household ownership of any form of smart technology, dogs, or cats. Adjusting the regressions for age, gender, and SES did not affect this. Table S2 in the Multimedia Appendix 1 lists full results on social cohesion.

**Figure 1.**
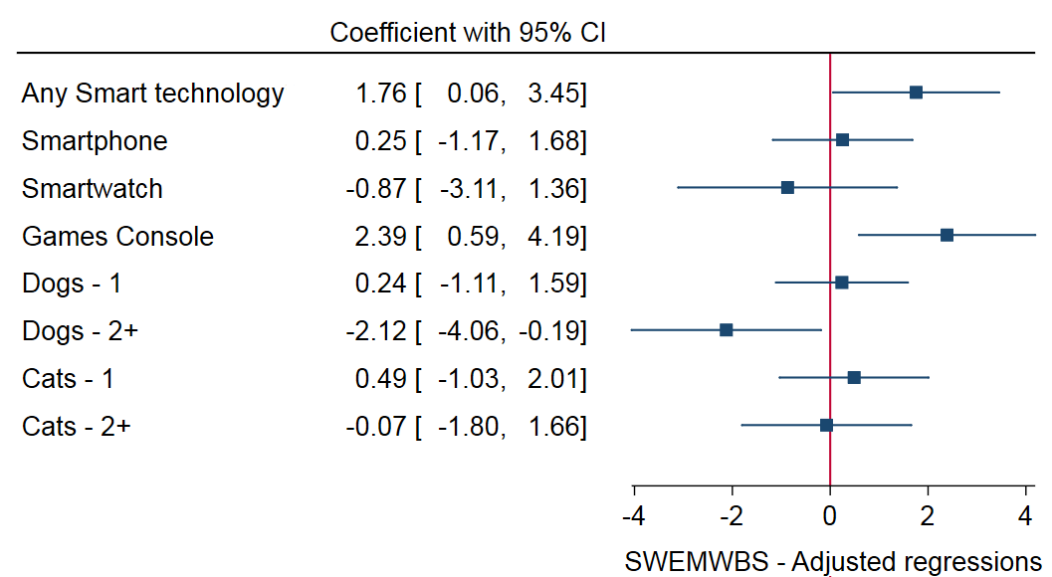
Mental well-being (SWEMWBS). Adjusted regressions (adjusted for age, gender, IMD decile 2015, education, employment, and social cohesion) for participating social housing residents in the Smartline cohort study in South West England, United Kingdom, 2017‐2018. IMD 2015: Index of Multiple Deprivation; SWEMWBS: Short Warwick-Edinburgh Mental Wellbeing Scale.

There was a significant difference in SWEMWBS scores by IMD 2015 ranking; being in the second or third decile was associated with lower SWEMWBS scores. Having studied in further education was also associated with significantly lower SWEMWBS scores. In unadjusted regressions, there was a significant effect of a household owning a smartphone or owning two or more dogs being associated with lower scores on SWEMWBS. The former effect did not hold after adjusting the model. However, owning two or more dogs remained associated with lower SWEMWBS scores after adjusting for age, gender, SES, and social cohesion. Adjustments were made to the other variables of interest. Owning any smart technology was significantly associated with higher SWEMWBS scores after adjusting for age, gender, and SES, and remained so after adding social cohesion to the model. Owning a games console was associated with higher SWEMWBS scores after adjusting for age, gender, and SES, and remained so after adding social cohesion to the model. There were no significant differences in SWEMWBS scores by cat ownership. Full results on SWEMWBS can be found in Table S3 in the Multimedia Appendix 1; the fully adjusted models are presented in Figure 1.

## Discussion

### Principal Findings

We found no evidence of increased social coherence among people with smart technology or pets among Smartline participants. This may reflect the relatively high level of uniformity in participants’ socioeconomic circumstances, and levels of social cohesion were more homogeneous than in White’s Welsh sample, with more than two-thirds of Smartline participants classified as having “medium” social cohesion. However, there was evidence that ownership of any form of smart technology, or a games console specifically, was associated with higher levels of mental well-being, while owning several dogs was associated with worse mental well-being. There were no associations for cat ownership.

Previous research on social cohesion and technology has had mixed results and appears to be heavily context dependent, and this may be what our neutral findings reflect. Good ICT can encourage stronger community connections, but it can also enable remote working and increased social isolation [10]. It can both exacerbate and bridge social divides [10,37], and it may prove more beneficial in places that already have a well-developed sense of local identity [38]. Miklian et al [37] suggest that smart technology is more usefully viewed as a medium, rather than as an entity which is inherently either positive or negative; it reflects the existing state of a community’s social cohesion, rather than enhancing or damaging it. Subsequent to the period of our data collection, research found that digital initiatives during the pandemic supported social networks [39]. However, this came with the caveat that those with limited technological skills needed pre-existing social connections to help them access such initiatives. Our findings challenge the notion that smart technology alone is sufficient to improve community circumstances.

Owning at least one form of smart technology allows some sociodemographic groups to maintain or improve their well-being. This may be through mechanisms such as social connectivity, accessing health advice, using well-being apps, or following exercise programs. Using specific well-being apps can improve positive affect and reduce depressive symptoms [40], and improve mental well-being [41,42]. Meanwhile, problematic smartphone use, defined as poor ability to self-limit smartphone use, has been negatively associated with family health [43]. Among UK adolescents, the use of digital technology has varied effects on mental well-being depending on factors including time of the week, type of activity, and level of use, and researchers conclude that overall the level of risk to mental well-being is modest [44]. We simply recorded household ownership and therefore could not explore issues such as the type and intensity of use further.

We found that owning a games console is associated with higher mental well-being among certain groups: one possible explanation is that the socially interactive aspect of gaming may have beneficial effects. While we are unable to substantiate this from our data, a review of research into young people’s use of video games suggests that, contrary to popular belief, gaming can have many beneficial effects on well-being, including emotional well-being, relationships, and social connections [45]. Furthermore, gaming can have cognitive benefits and improve people’s motivation [46], both of which may have downstream benefits to well-being. Research on gameplay during the pandemic found beneficial effects, including reduced stress and anxiety, social interaction, and cognitive stimulation [47]. However, our finding could also reflect the presence of children in the home and a degree of improved well-being arising from family support or may indicate a higher level of wealth. Our field notes indicate that at least 2 participants, one of whom had limited mobility, were using consoles for financial gain, for example, being paid to produce YouTube videos of gameplay, highlighting the potential role of consoles as a source of income and autonomy; an intriguing prospect for further exploration.

Research on dog ownership and social cohesion, although limited, suggests that dog ownership can contribute to neighborhood cohesion [17,23], although this was not supported by our data. Previous work suggests that social capital (engaging in activities with neighbors, experiencing trust, and reciprocity) has a limited [18] or no [48] association with dog ownership. In our data, owning 2 or more dogs was associated with lower levels of mental well-being: we hypothesize that owning several dogs places a burden on households in certain groups, whether in terms of time, finances, noise, or allergens: future research could explore this further. Our data were collected before COVID-19; however, the pandemic may have exacerbated such challenges. Many people acquired dogs for the first time during the pandemic, with less ability to preresearch their choice than in normal times, and less focus on canine health, potentially increasing the burdens of dog ownership [49]. Pets can replace human company, particularly for older people: Age UK reports that half of the people aged 65 and over in the United Kingdom rely on pets or television for companionship [50]. However, they may not replace human company adequately, so some people may own dogs specifically because they have poor mental health or are lonely [51]. Loneliness is a particular concern among older age groups, and further research is needed [52]. Parslow et al [53] also found negative effects of pet ownership among older Australians. Those with pets had higher rates of depression than those without, as well as worse physical health among women; they hypothesized that owning a pet meant carrying out more chores, and this effect would be multiplied with several pets. This may be particularly true among older age groups [54]. We also consider the possibility that owning a dog could reflect concerns about safety within a neighborhood, which could be explored explicitly in future studies.

We did not find any differences in mental well-being by cat ownership. There is limited research on cat ownership compared with dog ownership, and there have been calls to consider specific activities with cats, rather than simply “ownership,” in order to take account of potentially positive activities, such as play and tactile interactions, as opposed to those that may prove stressful, such as veterinary visits and illness [25].

Social housing organizations increasingly take on a role of supporting social interaction among residents [55,56]. Social cohesion during and after the pandemic was mediated by the efforts made by local organizations, with communities that invested in social cohesion experiencing better social outcomes [57]. Thus, communities living in social housing may have fared better.

### Limitations

Data were collected before the COVID-19 pandemic, which saw changes in perceived social cohesion, both positive and negative [57,58], as well as in pet ownership and use of smart technology. A low proportion of the sample reported currently being in work; this is likely to reflect the fact that recruitment took place during the day, when employed people were less likely to be home. It may also partially explain the low proportion of people reporting further or higher education and the relatively high mean age of the participants. We collected the main participants’ personal perceptions of social cohesion and their mental well-being; however, ownership of smart technology and pets related to the whole household; the main respondent may not have been the user of the technology or the main carer for the pet. We therefore could not control for further variables such as frequency or purpose of smart technology use, or of subjective relationships with pets: this would be an interesting avenue for further research. The sample size was modest, with some small cell sizes: it was not feasible to carry out further analyses, and the findings should be treated with caution. These cross-sectional data do not provide insights into the direction of any causality.

### Conclusions

Smart technology and ICT can have mixed effects on social cohesion and mental well-being and should not be treated as a panacea for community health. Among our sample of social housing residents in Cornwall, there was some evidence of associations between mental well-being and smart technology. Previous research suggests that dog ownership can have modest beneficial effects on health, well-being, and social interactions; our findings contradict this, showing that owning 2 or more dogs is associated with lower levels of mental well-being. People own smart technology and pets for different reasons, and they affect people’s lives in different ways, both good and bad. This may account for their mixed effects on social cohesion and well-being in this study, where positive and negative effects may have canceled each other out. Subsequent to this fieldwork, such effects may have been exacerbated by the COVID-19 pandemic [59]. Further research could clarify the question of who owns and uses a given form of technology within a household, and who takes care of a pet, to provide more nuance in analyzing psychosocial outcomes.

## Supplementary material

10.2196/75451 Multimedia Appendix 1 Further data for social cohesion, mental well-being, and the role of smart technology and pet ownership among social housing residents: a cross-sectional cohort study.

## Abbreviations

ICT: internet and communications technologies
IMD: Index of Multiple Deprivation
SES: socioeconomic status
STROBE: Strengthening the Reporting of Observational Studies in Epidemiology
SWEMWBS: Short Warwick-Edinburgh Mental Wellbeing Scale

## References

[1] O’Shea N, Bell A A spending review for wellbeing. briefing.

[2] Deeming C (2013). Addressing the Social determinants of subjective wellbeing: the latest challenge for social policy. J Soc Policy.

[3] Wallace C, Vincent K (2017). Handbook of Community Well-Being Research.

[4] Harrison EK, Quick A, Abdallah S (2016). Looking through the wellbeing kaleidoscope: results from the European Social Survey.

[5] (2018). Cornwall-UK. Steps towards a digital rural region.

[6] Williams AJ, Maguire K, Morrissey K, Taylor T, Wyatt K (2020). Social cohesion, mental wellbeing and health-related quality of life among a cohort of social housing residents in Cornwall: a cross sectional study. BMC Public Health.

[7] Thapa D, Sein MK, Sæbø Ø (2012). Building collective capabilities through ICT in a mountain region of Nepal: where social capital leads to collective action. Information Technology for Development.

[8] Broadbent R, Papadopoulos T (2013). Impact and benefits of digital inclusion for social housing residents. Community Dev (Columb).

[9] Stuart A, Yan RJ, Harkin L (2022). Design of a digital intervention to improve connectivity and reduce loneliness in older adults (preprint). JMIR Formative Research.

[10] Wallace C, Vincent K, Luguzan C, Townsend L, Beel D (2017). Information technology and social cohesion: A tale of two villages. J Rural Stud.

[11] Sehmer E As a child psychiatrist, I see what smartphones are doing to kids’ mental health – and it’s terrifying.

[12] Jonathan H (2025). The Anxious Generation: How the Great Rewiring of Childhood Is Causing an Epidemic of Mental Illness.

[13] Carter B, Ahmed N, Cassidy O (2024). “There’s more to life than staring at a small screen”: a mixed methods cohort study of problematic smartphone use and the relationship to anxiety, depression and sleep in students aged 13-16 years old in the UK. BMJ Ment Health.

[14] Kalk NJ, Downs J, Clark B, Carter B (2024). Problematic smartphone use: What can teenagers and parents do to reduce use?. Acta Paediatr.

[15] Hasan H, Linger H (2016). Enhancing the wellbeing of the elderly: social use of digital technologies in aged care. Educ Gerontol.

[16] Lavoie R, Zheng Y (2023). Smartphone use, flow and wellbeing: a case of Jekyll and Hyde. Comput Human Behav.

[17] Gómez E, Baur JWR, Malega R (2018). Dog park users: an examination of perceived social capital and perceived neighborhood social cohesion. J Urban Aff.

[18] Koohsari MJ, Yasunaga A, Shibata A (2021). Dog ownership, dog walking, and social capital. Humanit Soc Sci Commun.

[19] Cutt HE, Knuiman MW, Giles-Corti B (2008). Does getting a dog increase recreational walking?. Int J Behav Nutr Phys Act.

[20] White MP, Elliott LR, Wheeler BW, Fleming LE (2018). Neighbourhood greenspace is related to physical activity in England, but only for dog owners. Landsc Urban Plan.

[21] Costa S, Sousa L, Luz H, Padeiro M (2022). Daily mobility and social interactions among community-dwelling older adults with pet dogs: a scoping review. J Appl Gerontol.

[22] Wells DL (2004). The facilitation of social interactions by domestic dogs. Anthrozoös.

[23] Rogers J, Hart LA, Boltz RP (1993). The role of pet dogs in casual conversations of elderly adults. J Soc Psychol.

[24] Walsh F (2009). Human-animal bonds I: the relational significance of companion animals. Fam Process.

[25] Ravenscroft SJ, Barcelos AM, Mills DS, Banks J (2021). Cat-human related activities associated with human well-being. Hum Anim Interact Bull.

[26] Spitznagel MB, Gober MW, Patrick K (2023). Caregiver burden in cat owners: a cross-sectional observational study. J Feline Med Surg.

[27] Gosling SD, Sandy CJ, Potter J (2010). Personalities of self-identified “dog people” and “cat people”. Anthrozoös.

[28] van den Berg P, Sharmeen F, Weijs-Perrée M (2017). On the subjective quality of social Interactions: Influence of neighborhood walkability, social cohesion and mobility choices. Transportation Research Part A: Policy and Practice.

[29] Walton E (2018). The meaning of community in diverse neighborhoods: stratification of influence and mental health. Health Place.

[30] Menneer T, Qi Z, Taylor T (2021). Changes in domestic energy and water usage during the UK COVID-19 lockdown using high-resolution temporal data. Int J Environ Res Public Health.

[31] Buckner JC (1988). The development of an instrument to measure neighborhood cohesion. American J of Comm Psychol.

[32] Ng Fat L, Scholes S, Boniface S, Mindell J, Stewart-Brown S (2017). Evaluating and establishing national norms for mental wellbeing using the short Warwick-Edinburgh Mental Well-being Scale (SWEMWBS): findings from the Health Survey for England. Qual Life Res.

[33] Stewart-Brown S (2018). The warwick-edinburgh mental wellbeing scales - WEMWBS. Warwick Medical School.

[34] StataCorp (2019). Stata statistical software: release 16. https://www.stata.com/.

[35] von Elm E, Altman DG, Egger M, Pocock SJ, Gøtzsche PC, Vandenbroucke JP (2008). The Strengthening the Reporting of Observational Studies in Epidemiology (STROBE) statement: guidelines for reporting observational studies. J Clin Epidemiol.

[36] White J, Greene G, Farewell D (2017). Improving mental health through the regeneration of deprived neighborhoods: a natural experiment. Am J Epidemiol.

[37] Miklian J, Hoelscher K (2017). Smart cities, mobile technologies and social cohesion in India. Indian Journal of Human Development.

[38] Beel D, Wallace C (2020). Gathering together: social capital, cultural capital and the value of cultural heritage in a digital age. Social & Cultural Geography.

[39] Phillips RH (2024). The role of ICT in maintaining social cohesion: understanding the potential of digital initiatives for social networks in rural areas. Rural Sociol.

[40] Howells A, Ivtzan I, Eiroa-Orosa FJ (2016). Putting the ‘app’ in happiness: a randomised controlled trial of a smartphone-based mindfulness intervention to enhance wellbeing. J Happiness Stud.

[41] Bakker D, Rickard N (2019). Engagement with a cognitive behavioural therapy mobile phone app predicts changes in mental health and wellbeing: MoodMission. Aust Psychol.

[42] McEwan K, Richardson M, Sheffield D, Ferguson FJ, Brindley P (2019). A smartphone app for improving mental health through connecting with urban nature. Int J Environ Res Public Health.

[43] Guo N, Wang MP, Luk TT (2019). The association of problematic smartphone use with family well-being mediated by family communication in Chinese adults: a population-based study. J Behav Addict.

[44] Przybylski AK, Weinstein N (2017). A large-scale test of the goldilocks hypothesis. Psychol Sci.

[45] Johnson D, Jones C, Scholes L, Carras MC (2013). Videogames and wellbeing: a comprehensive review. https://eprints.qut.edu.au/105915/.

[46] Granic I, Lobel A, Engels RCME (2014). The benefits of playing video games. Am Psychol.

[47] Barr M, Copeland-Stewart A (2022). Playing video games during the COVID-19 pandemic and effects on players’ well-being. Games and Culture.

[48] Martin E (2024). The effects of dog ownership and social capital.

[49] Packer RMA, Brand CL, Belshaw Z, Pegram CL, Stevens KB, O’Neill DG (2021). Pandemic puppies: characterising motivations and behaviours of UK owners who purchased puppies during the 2020 COVID-19 pandemic. Animals (Basel).

[50] Davidson S, Rossall P (2015). Age UK evidence review: loneliness in later life. https://www.ageuk.org.uk/siteassets/documents/reports-and-publications/reports-and-briefings/health--wellbeing/rb_june15_lonelines_in_later_life_evidence_review.pdf.

[51] Müllersdorf M, Granström F, Sahlqvist L, Tillgren P (2010). Aspects of health, physical/leisure activities, work and socio-demographics associated with pet ownership in Sweden. Scand J Public Health.

[52] Bruggencate TT, Luijkx KG, Sturm J (2018). Social needs of older people: a systematic literature review. Ageing Soc.

[53] Parslow RA, Jorm AF, Christensen H, Rodgers B, Jacomb P (2005). Pet ownership and health in older adults: findings from a survey of 2,551 community-based Australians aged 60-64. Gerontology.

[54] Wells DL (2009). The effects of animals on human health and well‐being. Journal of Social Issues.

[55] Purkis A Housing associations in england and the future of voluntary organisations. The Baring Foundation.

[56] Blessing A (2012). Magical or monstrous? Hybridity in social housing governance. Hous Stud.

[57] Lalot F, Abrams D, Broadwood J, Davies Hayon K, Platts-Dunn I (2022). The social cohesion investment: communities that invested in integration programmes are showing greater social cohesion in the midst of the COVID-19 pandemic. J Community Appl Soc Psychol.

[58] Borkowska M, Laurence J (2021). Coming together or coming apart? Changes in social cohesion during the COVID-19 pandemic in England. European Societies.

[59] Mikolai J, Keenan K, Kulu H (2020). Intersecting household-level health and socio-economic vulnerabilities and the COVID-19 crisis: an analysis from the UK. SSM Popul Health.

[60] Woods RD, Menneer T, Wellaway I (2023). Smartline environmental sensor data and utility usage, 2017–2023. Smartline Environmental Sensor Data and Utility Usage.

