# Supplementary material for: Social Cohesion, Mental Well-Being, and the Role of Smart Technology and Pet Ownership Among Social Housing Residents: Cross-Sectional Cohort Study
Source: JMIR Form Res. 2025 Oct 24;9:e75451. doi: 10.2196/75451 (PMC12555080; doi:10.2196/75451)
Supplement: Multimedia Appendix 1 [file formative-v9-e75451-s001.pdf]

Appendix: Social cohesion, mental wellbeing and the role of smart technology and pet ownership among social housing residents: a cross-sectional cohort study

Table S1: Variable table providing a description of key variables used in analysis of social housing residents participating in the Smartline cohort study in South West England, UK, 2017 - 2018

VARIABLE	VARIABLE DESCRIPTION
**AGE**	Main participant's age in years at time of participation
**GENDER**	Main participant's gender
**IMD 2015**	Household's place in the decile rankings according to the Index of Multiple Deprivation, based on postcode
**RUC 2011**	Household's classification as "Urban city and town" or "Other" according to Rural Urban classifications, based on postcode
**EDUCATION**	Main participant's highest level of education
**EMPLOYMENT**	Main participant's employment status
**SOCIAL COHESION**	Main participant's perception of social cohesion in their neighbourhood, based on Buckner's Neighbourhood Cohesion Instrument
**SWEMWBS**	Main participant's perception of their own mental wellbeing, based on the Short Warwick-Edinburgh Mental Wellbeing Scale
**ANY SMART TECH**	Household ownership of *any* smart technology
**SMARTPHONE**	Household ownership of a smartphone
**SMARTWATCH**	Household ownership of a smartwatch
**GAMES CONSOLE**	Household ownership of a games console
**DOGS**	Household ownership of dogs, and number
**CATS**	Household ownership of cats, and number

IMD 2015: Index of Multiple Deprivation, RUC 2011: Rural Urban Classification 2011, SWEMWBS: Short Warwick-Edinburgh Mental Wellbeing Scale.

Table S2: Univariable associations for Social Cohesion and Mental Wellbeing (SWEMWBS) for social housing residents participating in the Smartline cohort study in South West England, UK, 2017 - 2018

		SOCIAL COHESION		SWEMWBS	
		Coef	[95% CI]	Coef	[95% CI]
**AGE**	Years	<-0.01	[-0.04, 0.04]	0.08	[0.04, 0.11]
**GENDER**	Female	ref		ref	
	Male	0.77	[-0.80,2.34]	-0.02	[-1.29,1.26]
**IMD 2015**	ranking	<-0.01	[<-0.01, <0.01]	<-0.01	[<-0.01, <0.01]
	1st decile (most)	ref		ref	
	2nd decile	-0.45	[-2.57,1.68]	-1.72*	[-3.44,-0.01]
	3rd decile	-0.37	[-2.68,1.93]	-2.20*	[-4.06,-0.34]
	4th decile	-1.30	[-3.14,0.54]	-0.87	[-2.35,0.61]
	5th decile	-	-	-	-
	6th decile	-	-	-	-
	7th decile	-	-	-	-
	8th decile	-	-	-	-
	9th decile	-	-	-	-
	10th decile (least)	-	-	-	-
**RUC 2011**	Urban city and town	ref		ref	
	Other	-0.01	[-2.88,2.87]	0.24	[-2.10,2.58]
**EDUCATION**	Secondary and/or primary	ref		ref	
	Further	-0.69	[-2.32,0.94]	-1.86**	[-3.17,-0.54]
	Higher	1.48	[-1.81,4.78]	-0.61	[-3.27,2.04]
**EMPLOYMENT**	In work	1.05	[-0.91, 3.02]	1.48	[-0.06, 3.02]

	Education or training			1.95	[-2.97, 6.87]	0.45	[-3.41, 4.31]
	Retired			1.27	[-0.39, 2.93]	3.38	[2.08, 4.69]
	Not in work		ref			ref	
**ANY SMART TECH**	No		ref			ref	
	Yes			0.77	[-1.28,2.82]	0.25	[-1.42,1.92]
**SMARTPHONE**	No		ref			ref	
	Yes			0.06	[-1.52,1.65]	-1.39*	[-2.67,-0.11]
**SMARTWATCH**	No		ref			ref	
	Yes			0.99	[-1.77,3.74]	-1.31	[-3.55,0.93]
**GAMES CONSOLE**	No		ref			ref	
	Yes			0.91	[-1.37,3.19]	1.37	[-0.48,3.22]
**DOGS**		0	ref			ref	
		1		-1.3	[-3.02,0.42]	-0.43	[-1.82,0.96]
	2+			0.96	[-1.52,3.44]	-2.84**	[-4.84,-0.83]
**CATS**		0	ref			ref	
		1		-0.15	[-2.12,1.82]	0.03	[-1.57,1.63]
	2+			1.32	[-0.85,3.49]	-0.52	[-2.29,1.25]

Table S3: Adjusted associations for Social Cohesion and Mental Wellbeing (SWEMWBS) (adjusted for age, gender, IMD decile 2015, Education, Employment; and Social Cohesion) for social housing residents participating in the Smartline cohort study in South West England, UK, 2017 – 2018. *p<.05 **p<.01 ***p<.001.

		**SOCIAL COHESION**<br><br>**ADJUSTED FOR AGE, GENDER, IMD DECILE 2015, EDUCATION, EMPLOYMENT**		**SWEMWBS**<br>**ADJUSTED FOR AGE, GENDER, IMD DECILE 2015, EDUCATION, EMPLOYMENT**		**SWEMWBS**<br>**ADJUSTED FOR AGE, GENDER, IMD DECILE 2015, EDUCATION, EMPLOYMENT AND SOCIAL COHESION**	
		Coef	[95% CI]	Coef	[95% CI]	Coef	[95% CI]
**ANY SMART TECH**	No	ref		ref		ref	
	Yes	1.17	[-1.09,3.43]	1.99*	[0.24,3.75]	1.76*	[0.06,3.45]
**SMARTPHONE**	No	ref		ref		ref	
	Yes	0.65	[-1.24,2.54]	0.39	[-1.09,1.87]	0.25	[-1.17,1.68]
**SMARTWATCH**	No	ref		ref		ref	
	Yes	0.74	[-2.23,3.71]	-0.72	[-3.03,1.60]	-0.87	[-3.11,1.36]
**GAMES CONSOLE**	No	ref		ref		ref	
	Yes	1.05	[-1.36,3.47]	2.60**	[0.74,4.46]	2.39**	[0.59,4.19]
**DOGS**	0	ref		ref		ref*	
	1	-1.23	[-3.02,0.56]	-0.03	[-1.43,1.37]	0.24	[-1.11,1.59]
	2+	1.23	[-1.35,3.80]	-1.86	[-3.86,0.15]	-2.12*	[-4.06,-0.19]
**CATS**	0	ref		ref		ref	
	1	0.03	[-1.99,2.04]	0.49	[-1.08,2.07]	0.49	[-1.03,2.01]
	2+	1.46	[-0.82,3.75]	0.23	[-1.55,2.02]	-0.07	[-1.80,1.66]
